# Visualization of data in radiotherapy using web services for optimization of workflow

**DOI:** 10.1186/s13014-014-0322-3

**Published:** 2015-01-20

**Authors:** Stefan Kirrmann, Mark Gainey, Fred Röhner, Markus Hall, Gregor Bruggmoser, Marianne Schmucker, Felix E Heinemann

**Affiliations:** Klinik für Strahlenheilkunde, Department für Radiologische Diagnostik und Therapie, Universitätsklinikum Freiburg, Robert Koch Str. 3, 79106 Freiburg, Germany

**Keywords:** Clinic management, Web-services, Information management, Workflow, Quality management, Radiotherapy

## Abstract

**Background:**

Every day a large amount of data is produced within a radiotherapy department. Although this data is available in one form or other within the centralised systems, it is often not in the form which is of interest to the departmental staff. This work presents a flexible browser based reporting and visualization system for clinical and scientific use, not currently found in commercially available software such as MOSAIQ^TM^ or ARIA^TM^. Moreover, the majority of user merely wish to retrieve data and not record and/or modify data. Thus the idea was conceived, to present the user with all relevant information in a simple and effective manner in the form of web-services. Due to the widespread availability of the internet, most people can master the use of a web-browser. Ultimately the aim is to optimize clinical procedures, enhance transparency and improve revenue.

**Methods:**

Our working group (BAS) examined many internal procedures, to find out whether relevant information suitable for our purposes lay therein. After the results were collated, it was necessary to select an effective software platform. After a more detailed analysis of all data, it became clear that the implementation of web-services was appropriate. In our institute several such web-based information services had already been developed over the last few years, with which we gained invaluable experience. Moreover, we strived for high acceptance amongst staff members.

**Results:**

By employing web-services, we attained high effectiveness, transparency and efficient information processing for the user. Furthermore, we achieved an almost maintenance-free and low support system. The aim of the project, making web-based information available to the user from the departmental system MOSAIQ, physician letter system MEDATEC^R^ and the central finding server MiraPlus (laboratory, pathology and radiology) were implemented without restrictions.

**Conclusion:**

Due to widespread use of web-based technology the training effort was effectively nil, since practically every member of staff can master the use of a web-browser. Moreover, we have achieved high acceptance amongst staff members and have improved our effectiveness resulting in a considerable time saving.

The many MOSAIQ-specific parts of the system can be readily used by departments which use MOSAIQ as the departmental system.

## Background

The processes and procedures within a radiotherapy department are complex and correlations can only be determined and understood with considerable effort. An appropriate processing of incoming data, and its visualization, appears in this context very judicious and it is/was the aim of this project. Up to 250 patients are irradiated daily within our department, approximately 3000 patients are treated annually and 4500 patients receive follow-up care on an outpatient basis.

In radiotherapy there are two commercial departmental systems available (ARIA from Varian and MOSAIQ from Elekta). All systems have a reporting tool either integrated or available as an add-on package. However they do not adequately support the requirements as currently implemented in this project. In addition, clinic-wide information systems, such as ISH-Med from SAP, offer reporting capabilities. However, these too does not meet the special requirements of modern radiotherapy.

Alternatively, Citrix does indeed offer a partial solution that renders (local) installation of software on computers redundant. However, if one were to use Citrix to perform remote access, one would nevertheless have to develop special tools to run remotely on the Citrix server, to achieve the same functionality. Thus, in the opinion of the authors, although the Citrix solution may be interesting for small to medium sized radiotherapy departments, it is not a viable solution in our clinic with over 140 workstations. Citrix remains interesting for radiotherapy practices which are located at different sites.

There are many reporting systems currently available such as Crystal Reports, Aqua Data Studio and so on, for extracting and presenting data. We also use these tools in the context of our development, for example to optimize database queries. However, these reporting systems are not suitable for a user-friendly and user-centric working environment.

Moreover, Elekta provides a scripting interface (WFM), which is currently in the development phase. We employ this add-on for some problems. Similarly in relation to this Varian is in the development phase. The scripting is not able to provide us with key features implemented in our project. It is of course our aim to implement these features with the scripting interface in the future.

After incorporating the organisational model of treatment planning into our departmental system MOSAIQ [1,2], a complete document management system was embedded in our digital environment [3]. In our clinical centre a central findings server (MiraPlus) was set-up, within the context of the higher-ranking Hospital Information System (HIS), upon which all finding data (Laboratory, Pathology, diagnostic Radiology and so on) of the University Clinic are to be found [4-6]. Storing finding data from the HIS in several departmental systems is largely redundant and difficult to handle due to changes, deletions and so on. It is only possible to access this data in accordance with specified rules (data protection). With in-house developments we were able to present radiotherapy relevant information from the HIS. If one wants to retrieve data from commercial systems, the disclosure of their data structures and the read-only permission to the respective database, are pre-requisites to establish programs to visualize all department relevant data. Furthermore many popular database systems have to be capable of being accessed via drivers. Moreover, this access is neither sensitive nor time-critical and read-only. Changes to the database are only performed either with the departmental system (MOSAIQ) or in the case of HIS, via its own applications. Multi-access to the database is controlled. Data derived from our own development are incorporated exclusively via the interface defined by the manufacturer.

Every day a large amount of data is produced within a radiotherapy department [7]. Although this data is available in one form or other within the centralised system, it is rarely in the form which is of interest to the user (for example auto-refreshing ward-list of patient appointments for the daily treatments).

Additionally, the appropriate programs (clients) must either be installed and maintained on all workstations, or on a Citrix server. Regardless of the installation mode one must nevertheless provide training. Moreover the majority of user merely wish to retrieve data and not record and/or modify data.

Thus the idea was conceived, to present the user with all relevant information in a simple and effective manner. Ultimately, the aim is to optimize clinical procedures, enhance transparency and improve revenue.

## Material and methods

**Figure 1:**
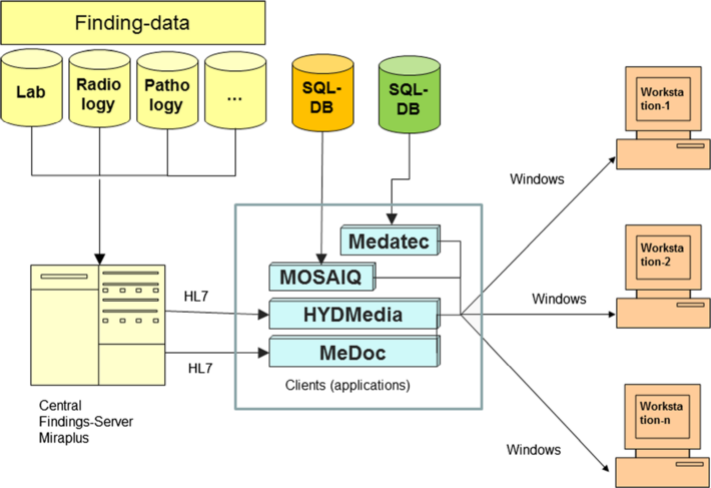
**Retrieval of data with clients.**

In order to achieve an optimal result, the requirements of the users within our clinic were analysed and documented. After the results were collated, the selection of the appropriate tools and platforms followed. In addition, clear criteria were discussed and defined in setting up the project. In Table 1 the selection criteria such as platform independence, parameterization, use of standards and so on are shown. The aim was to develop a control and retrieval system for all necessary radiotherapy related information, which above all is easy to use, highly effective and incorporates unproblematic handling. In Figure 1 the classic method, which is still the most common manner of data processing and visualization platforms in clinics, is shown [8]. This form of data processing occurs in practically all systems that record, manage and present data. The disadvantages of this mode of operation have already been mentioned above (Background). Furthermore, in order to use these programs time intensive teaching is required. This makes the maintenance and operation of the system laborious for the system administrator and user.

**Table 1:** **Selection criteria**

**Criterion**	**Description**
Platform-independent	The created programs have to be available to all popular platforms such as Linux, Windows and OSX
Parameterization	Hereby modification to programs shall be simplified and fast response to desired modification can be attained, whilst simultaneously reducing susceptibility to errors
Standards	Thus one achieves protection of the investment, good availability of the programs and transparency
Modularity	Construction of a toolbox und conformity of programming in modular form through specification of standards
Operational safety	Central webserver: Linux or Windows
User-friendliness	Achieved through implementation of standards and use of established structure design
Minimal training effort	Increased acceptance and performance, cost reduction
Effectivity	Functionality shall support and accelerate procedures
Transparency	Clearly understood functions for the user
Simplicity	Only functions which are regularly used
User-requirements	Simple and fast data access, group based permissions and templates

**Figure 2:**
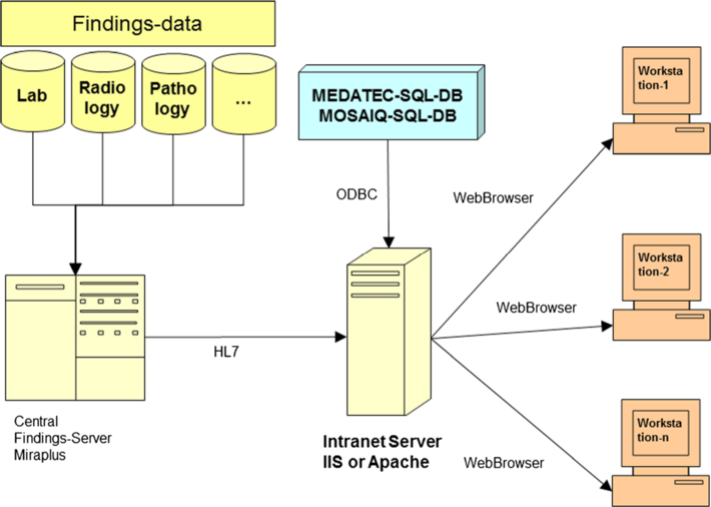
**Retrieval of data with web technology.**

**Figure 3:**
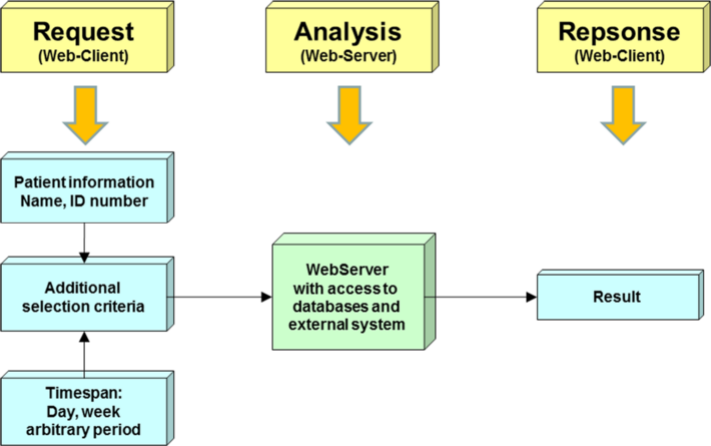
**Data selection process.**

In contrast the method schematically depicted in Figure 2, shows the standard web-services [9-11]. This browser technology is available on all major operating systems and enjoys high acceptance due to both its widespread use and mature status. The usage of common web-browsers such as Internet Explorer, Firefox and Chrome is simple and can be used by the majority of people without major teaching effort. The implementation of this platform normally requires only server-side activities of the IT-department, if for example new functionality is to be incorporated. A disadvantage is that complex procedures and controlling can only be realized with web-based technology [12-15] through considerable effort, particularly if they have to run modally. Despite this, they may still be more incident prone than other programming techniques. The former are, however, gaining in importance. They are excellent for editing, processing and displaying simple data structures, especially since diverse creation tools and libraries are currently available (interface functionality such as connection to databases, simple database queries and basic design of data presentation). After consideration and appraisal of all conditions and criteria, we decided therefore for a web-based system. We selected Linux and Windows 2003/2008 R2 as operating systems. Apache (Linux) and Internet Information Server (IIS) were selected as web-servers. The scripting languages PHP, Python, Ruby and Javascript were used as programming languages. The selection for data storage was reduced to MySQL, PostgreSQL (Linux) and MS SQL (Windows). In Figure 3 the structure of such an information system is depicted. After formulation of the selection criteria, a query is sent to the appropriate database which, after processing, returns a result to the requesting browser. The communication with the other systems uses HL7, DICOM, ODBC and proprietary interfaces. The programs were designed so that all data returned to the requesting system were online, up to date and dynamic (DHTML). At the first stage we realised the interface via HL7, since the majority of medical systems communicate via this Portal, so that, quantitatively, the largest data pool was accessible and that our entire HIS (PDV-FR, Findings Server MiraPlus) could be interrogated. Subsequently the provision of treatment or irradiation data (MOSAIQ) was achieved from the SQL-Database of the departmental system MOSAIQ via ODBC or a direct database driver. There were no standard interfaces available to format data from the physician’s letter system (MEDATEC). Therefore a proprietary interface had to be designed and programmed.

### The programming environment

**Figure 4:**
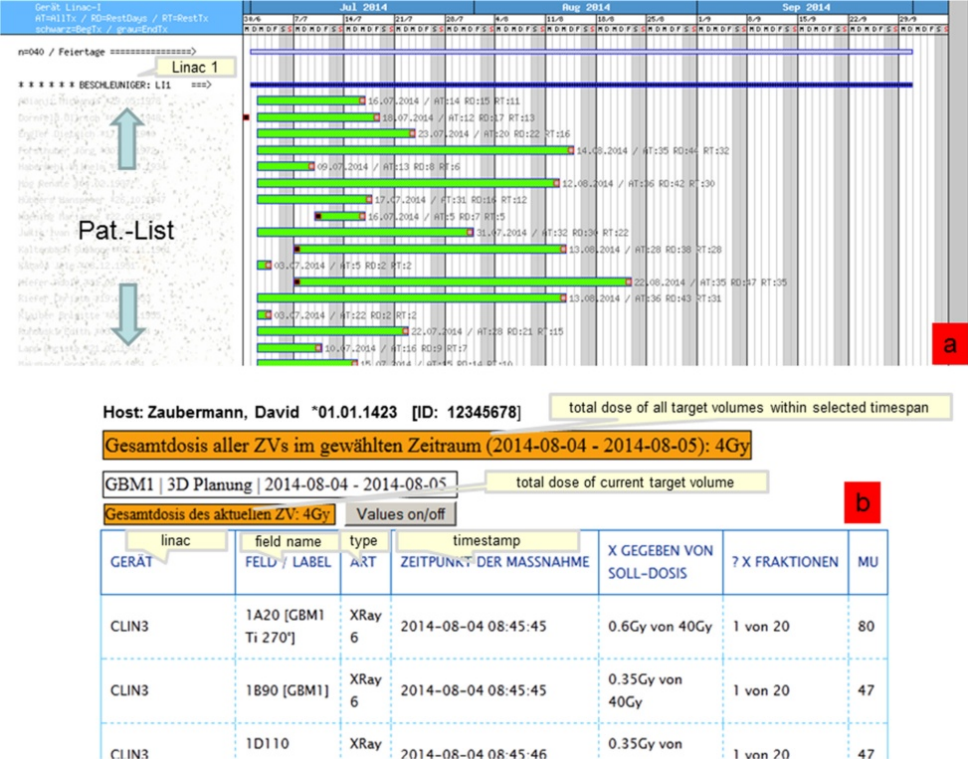
**Treatment data. a.** Working list of treatment machine with provisional treatment duration. **b.** Detailed treatment data for a patient such as field name, MU and so on.

The programming environment comprises a large number of modules and was mostly realised with PHP, Python, Ruby and Javascript including JQuery, as previously alluded to. For the communication via HL7, a special ISO-TCP protocol unique to our clinical centre, had to be represented (we provide standard TCP too), which was implemented in C++ at the first stage and later directly in PHP. A high degree of modularity was ensured through the construction and use of a PHP library specially created for this project. This library is continually being extended. After completion of the analysis and planning phase (approximately 1.5 months) and an intensive 3 month programming phase, 7 years ago, a first alpha test was completed, in which the fundamental functions were verified and revised (1 month), for example interface functionality such as connection to databases, simple database queries and basic design of data presentation . In the following beta test we sought out experienced staff members as test users and allowed them to work with the web-services for one month. As a result of which several essential changes were identified and implemented within this phase. Small modifications were also collected and in regular intervals implemented. Over the years a comprehensive collection of approximately 40 web-services (distributed over the data areas listed in Table 2) has arisen, which are all in routine use. New functions were always planned in accordance with the aforementioned method: programmed, evaluated and released. Since the calculation and depiction of irradiation data are very critically valued by the authors, we have therefore decided to limit the availability to simple treatment data (Figure 4b) such as field names and MUs.

**Table 2:** **Information portal**

**Data area**	**Data source**	**Description**
Intranet	Radiotherapy	General information portal with diverse links to department specific information
MOSAIQ	Radiotherapy	Information portal for all relevant data from our departmental system
MiraPlus	HIS	Portal to retrieve laboratory, pathology and radiology findings, including X-ray images
MEDATEC	Radiotherapy	Retrieval portal for all radiotherapy physicians’ letters since 1990
StK-Controlling	Radiotherapy	Retrieval of administrative and controlling data such as bed occupancy, case-mix, case data, linac workload, numbers of different, in particular special, therapy modes (Stereotaxy,HDR,LDR,IORT,IMRT,VMAT)
PDV-FR	HIS	Demographic patient data, case data, diagnosis and therapy (including appropriate ICD and OPS codes) of the HIS system
Request portal	Radiotherapy	Reading in of image data for treatment planning, support requests for IT (hardware, software problems, general user assistance for all IT and workflow question) etc.
Miscellaneous	HIS/Radiotherapy	Ward occupancy list with links to departmental system and ddepartment-controlling

At the current time the most important features have been implemented in a manner suitable for routine use. This is the primary reason why we have decided to publish this work now. In parallel we are implementing/developing design improvements, modules for general scientific problems and medical controlling for intra-departmental use.

The authors are continuing to work together with the manufacturer of our departmental system as part of an international working group, on the implementation of a workflow manager (WFM), which when verified will enable the depiction of sensitive irradiation data with web-services. The user has comfortable access to all program functionality via a standard Internet – browser (Figure 3). The installation of certain so-called plugins is not necessary since we strictly employ standard browser features (for example JavaScript) to implement the functionality.

### The information portal

Table 2 shows the individual data areas which are made available to the user. As one can clearly see, information is processed from all available systems. Thus, we enable the possibility to answer almost all questions with respect to information management.

In the following two examples will be described which emphasise the advantages and effectiveness of this projects; the depiction of all functionality is beyond the scope of this work.

### Ward occupancy portal

The ward occupancy portal serves as our first example with links to (irradiation) treatment appointments. In this tool the ward occupancy data from the HIS is linked with the treatment appointments from the MOSAIQ database. Hitherto the roll-out of this functionality, it was necessary for RTTs (linac radiographers) and ward staff to frequently check for changes to each patient undergoing radiotherapy appointment data. This led to friction, loss of time, and stress of the staff member concerned. After the implementation of the system, it was possible for the ward staff to retrieve the information (i.e. at what time a patient should be brought to a particular linac/afterloader) via a chronologically sorted ward/treatment list quickly and efficiently. Telephone calls have been rendered largely superfluous. Training for these applications was limited to ward staff, performed within 5 minutes and passed on to other members of staff by those initially trained; the user side installation consisted of the simple configuration of a web-link on the desktop computers on the ward. After careful consideration, installation of external programs and hardware was deemed undesirable. When one considers the case that our wards have their own IT-structure, with appropriate profiles and limitations, web-services was the only viable option to establish the functionality.

### Request portal

**Figure 5:**
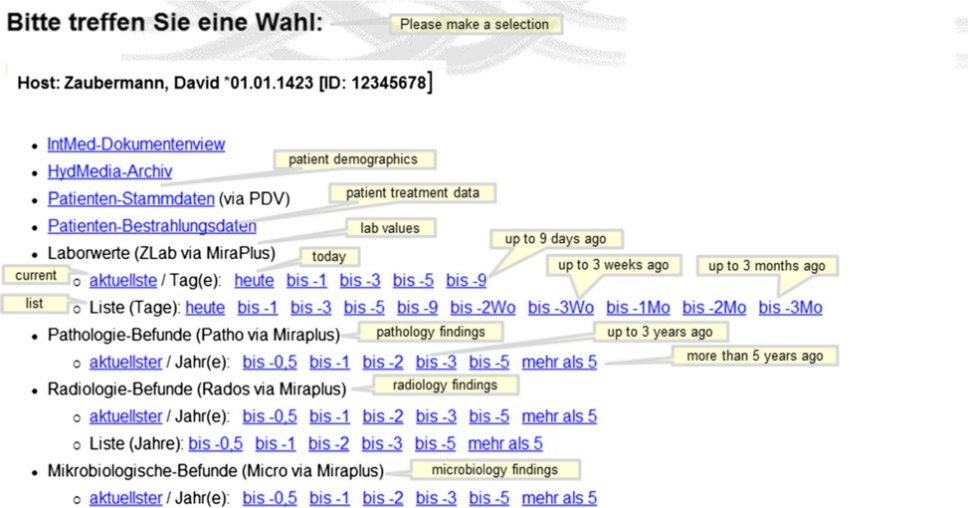
**Start page for findings and data retrieval.**

**Figure 6:**
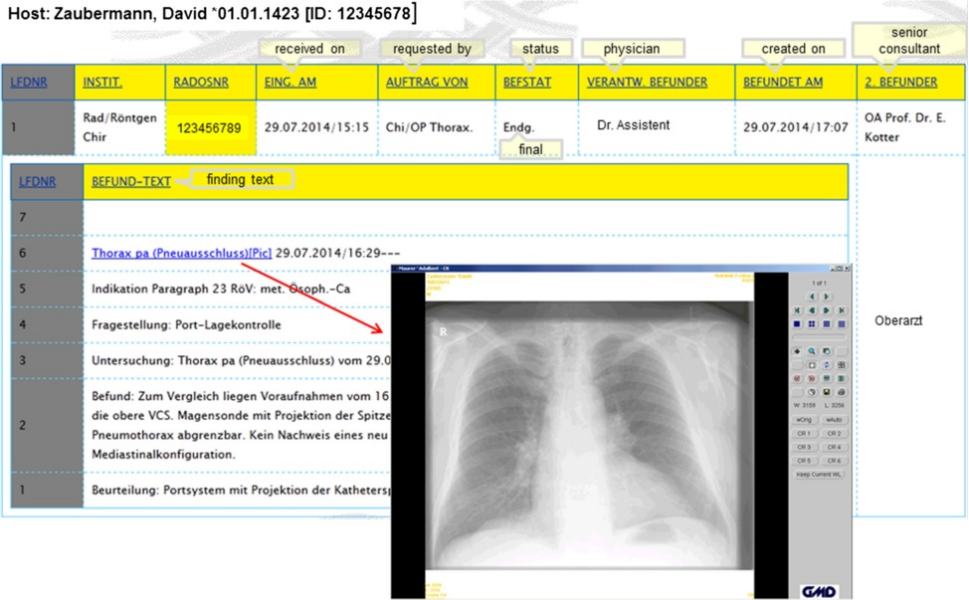
**Diagnostic x-ray findings/typical thoracic x-ray.**

**Figure 7:**
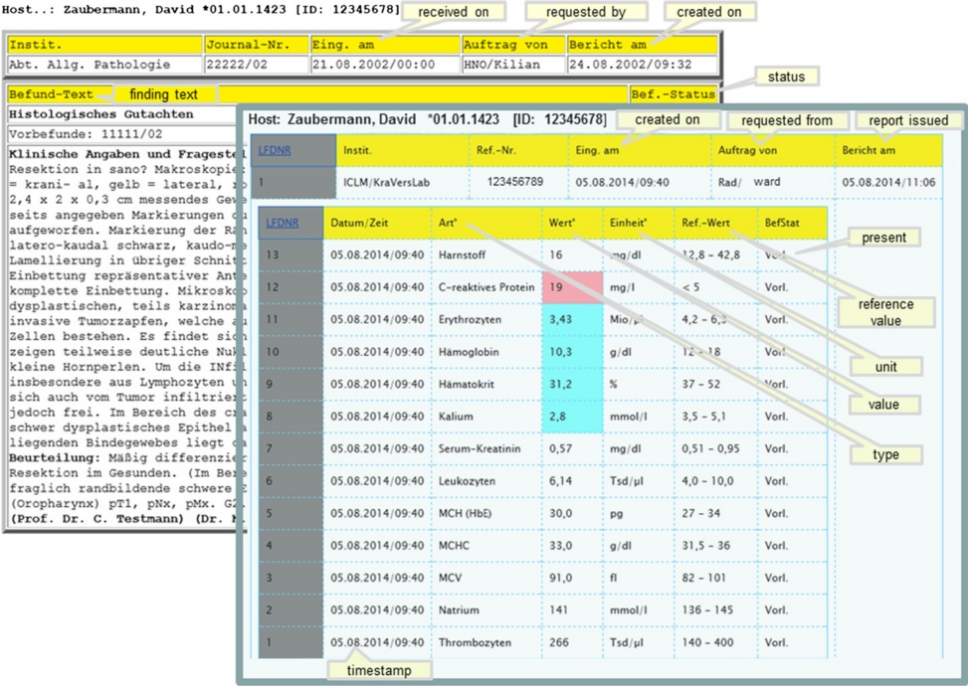
**Pathology and laboratory findings.**

**Figure 8:**
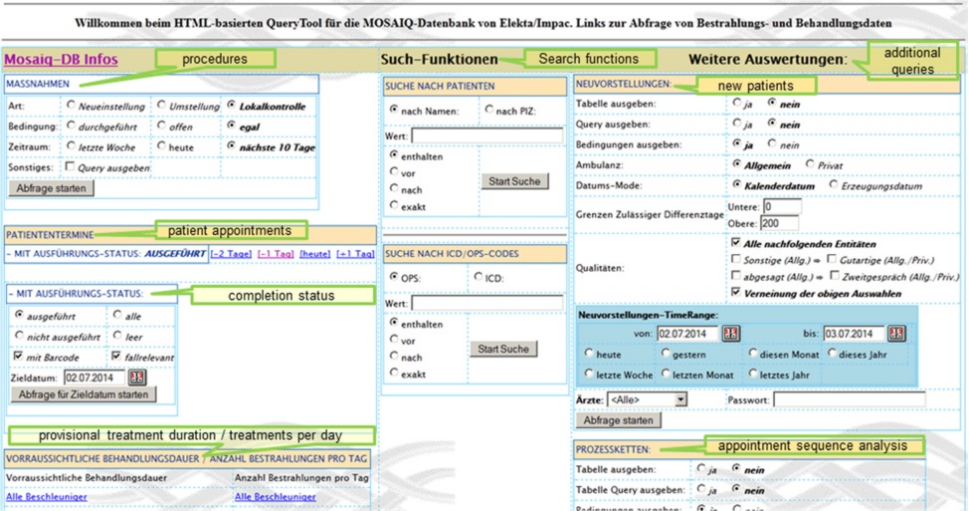
**Extract from the start page for the (irradiation) treatment data.**

**Figure 9:**
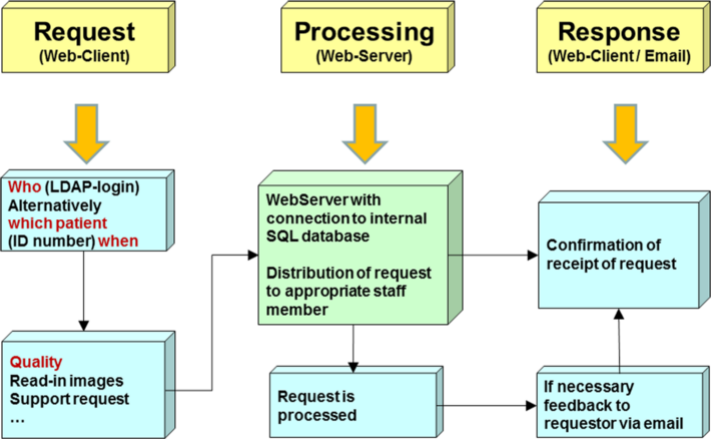
**Principle of operation of the request portal.**

**Figure 10:**
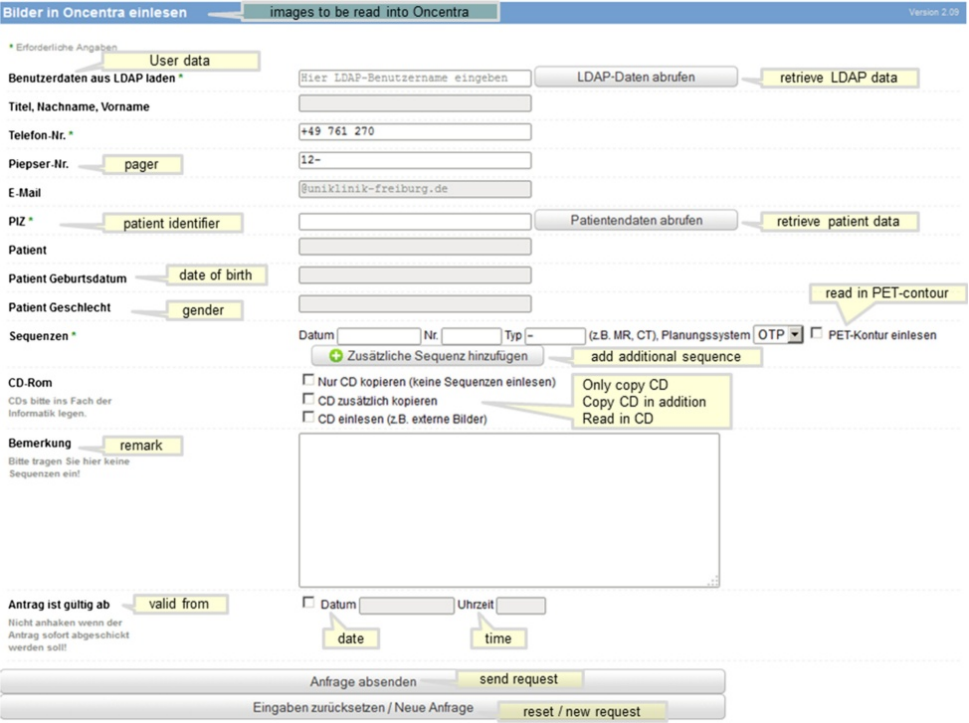
**Representative screenshot of the request portal.**

This web application contains many unique functions or requests with different usage profiles. In Table 3 we have summarized the most important functions of this portal. From the start page one arrives at a findings selection through a few mouse clicks, from which any finding can be retrieved (Figure 5). At this point we reiterate that most data would be online, current and dynamically edited, thus the user has up to date information available. Processing and depiction of other findings such as diagnostic x-ray, pathology and laboratory information are shown in Figures 6 and 7. Figure 6 shows an x-ray finding including a hyperlink to the image data. One navigates to the appropriate image by means of a single mouse click. Structured histopathology findings are also displayed with only a few mouse clicks. Even laboratory data with specified min and max values and depiction of pathology values (red = too high, blue = too low) are effortlessly displayed, see Figure 7. The processing and depiction of irradiation relevant data is of particular importance in our clinic [16,17]. There are even web-services which provide data to staff members for established treatment techniques such as intensity modulated radiotherapy (IMRT, VMAT), image guided radiotherapy (IGRT) for example by means of Cone Beam CT (CBCT) [18-20], in addition to special treatment forms such as stereotactic irradiation [21,22]. For example for IMRT/VMAT treatments field names, energy, dose contribution (as stored in MOSAIQ), MUs and current fraction number are displayed, this information is automatically updated at intervals of approximately 10 seconds. This tool is particularly useful for duty physicists or physicians who are legally required to remain within the department whilst patients are being irradiated, for example after repair or during routine service of a linac. A whole slew of depictions assist the medical, technical and nursing staff in their daily routine work, although pure administrative tasks are in the foreground. In the following Figures 4, 8, the visualization possibilities are representatively depicted. Figure 8 shows the selection window through which depictions of data, such as that found in Figure 4, can be attained. Currently approximately 40 different data groupings with distinct graphical display modes are offered, such as a Gantt-chart depicting the duration of (irradiation) treatment duration, tables for individual treatment parameters, and lists for simple text visualization. Of course the requirements and wishes of the users are continually being evaluated and integrated. Each user or user group selects (via a bookmark function) the views or functionalities which are important to him/her. Of course, the entire range of views is available to department staff via the (clinic) Intranet. The web-based request portal assists clinic staff mainly in organisational work in practically all tasks. The majority of routine tasks can be simply and elegantly performed with this tool packet. In Figure 9 the principle of operation and a representative screenshot (Figure 10) are shown. Due to simple operation the hitherto unsuccessful and repeated telephone calls to the responsible staff member are completely obsolete. The person making the request now has to consider only what information is required and not question such as “Who is (currently) responsible?”, “How can I contact this person?”

**Table 3:** **Request portal modules**

**Function**	**Description of the request**
Read Image data	Reading in image data from different sources for treatment planning
Anonymise image data	Handling of image data for research
IT-Support	General Technical IT-support:
	Hardware, software, network problems
	user assistance for all IT and workflow questions
Printer support	Support for all common printer issues and maintenance
Electronic door locking system	Exchange low batteries of the electronic door locking system
IORT appointments	Order/entry for IORT appointments

### Medicine controlling portal

This web-based portal presents a plurality of department specific analyses, which decisively contribute to a smooth and low error operation in our clinic. In addition these analyses contain economic and scientific information. In combination with administrative data we have an accurate and efficient view of our clinic. Moreover we can react promptly and develop ourselves further. The scope of this package is very large and, thus, will be described in a separate publication in the near future.

## Results/Discussion

The solution naturally comprises component parts typical for our department, for example the implementation of an ISO-TCP interface to the HIS. We can and indeed do also provide a standard TCP interface. Since there are very many MOSAIQ installations worldwide, the majority MOSAIQ-specific web-services are of interest to MOSAIQ users and simple to implement. Moreover, interfacing to other HIS systems is possible due to the modular construction of the component parts.

In principle we can make our web-services available to external users but only within the framework of cooperation, since we are not a commercial organization and cannot provide support. At the present time we cooperate with another clinic and a private practice (both MOSAIQ users).

Our approach has primarily focused on demonstrating to other radiotherapy departments, and above all the manufacturers (such as Elekta and Varian), how one can work better, beside the improvement in efficiency within our department. Moreover, we try to persuade the software producers to integrate our developments into their products, or to develop commercial products with partners. This approach has proven successful in the past, for example the automatic forwarding of services such as treatment planning see references or Classy Profile data extraction from the MOSAIQ digital patient file in cooperation with KHP.

In addition we organize meetings whose focus are the problems associated with clinical and administrative IT specific to radiotherapy. As part of this meeting we regularly provide a demo of our developments, including web-services. These demos are always booked out.

The visualization of departmental data via web-services presents an important and expedient addition to routine work in a radiotherapy clinic. The user is very effectively assisted in his work by our system. By implementing web services and organizational improvements, resulting from this work, we have increased the efficiency of our clinic considerably.

In our BAS-working group the modules are discussed and evaluated frequently. Checks of the log-files of the Webserver provide information concerning the use of each web-service. However, the most important aspect is the continuous dialogue with our staff.

Analysis of key data such as ward occupancy, CMI, DRGs and patient numbers from the view point of different diagnosis, therapy techniques and revenue situation help us to maintain our performance in daily clinical routine.

It has been demonstrated that not only have the selection of tools, the design and modularity decisively contributed to the effectiveness of the system, but also simplicity, adherence to widespread standards and low maintenance have resulted in a high degree of acceptance amongst users and administrators. The system has now been in operation for 7 years and is continually being extended. Despite the migration to a new departmental system (VISIR to MOSAIQ), the porting of existing web-applications was implemented without major difficulties. Our training and user support effort is vanishingly small and due to the use of browser technology updates can be performed considerably easier (generally only central, server side) compared to client-based applications. Additionally, the number of licences for applications is reduced since a considerable number of users retrieve data exclusively with web-services.

## Conclusions

It was possible to clearly demonstrate an optimization of processes, an increase of transparency and a reduction in redundancy by means of:

1. Access frequency (log-files) to the modules
2. Continuing dialogue with users
3. Evaluation of the modules by the BAS-working group
4. Analysis of key data such as ward occupancy, CMI, DRGs and patient numbers from the view point of different diagnosis, therapy techniques and revenue situation.

Moreover, since the introduction of the system the revenue continues to rise.

This project led to all relevant data being available at all times in a simple manner, from all computers irrespective of which component part they are derived from. This leads to improved efficiency and considerable time saving.

The many MOSAIQ-specific parts of the system can be readily used by departments which use MOSAIQ as the departmental system.

### Glossary of terms used in figures

- ausgeführt/durchgeführt - completed
- nicht ausgeführt - incomplete
- mit - with
- Fallrelevant - relevant to case
- enthalten - contains
- vor - before
- nach - after/according to
- exakt - exact
- Tabelle ausgeben - show table
- Query ausgeben - show query
- Bedingungen ausgeben - show conditions
- Ambulanz - ambulance
- Datums-Mode - date type
- Grenzen zulässiger Differenztage - permitted date limitation
- Qualitäten - qualities
- ja - yes
- Nein - no
- Allgemein - general
- privat - private
- Kalenderdatum - calendar date
- Erzeugungsdatum - creation date
- leer - empty
- Neueinstellung - first fraction
- Umstellung - change of treatment plan
- Lokalkontrolle – local control (weekly routine check of patients during radiation course)
- Art - type
- Bedingung - condition
- Zeitraum - timespan
- Sonstiges - other
- heute - today
- Letzte Woche - last week
- Nächste 10 Tage - next 10 days
- offen - Open (not completed)
- egal - irrelevant
- Zieldatum - date specification

## Abbreviations

Afterloader: Device for the brachytherapy
ARIA: Departmental system (Varian) R&V-System
BAS: Betriebs-Ablauf-System (process oriented System)
C++: Programming language
ConeBeam/CBCT: Digital volume tomography
DHTML: Dynamic use of HTML
Elekta: Vendor of accelerators radiotherapy software, R&V-System
HL7: Health Level Seven (standard for exchanging information between medical applications)
HTML: Hyper Text Markup Language
ICD: International classification of disease
IGRT: Image Guided Radiation Therapy
IMRT: Intensity Modulated Radiation Therapy
IntMed: Medatec html document viewer
Int-Tec: Vendor of software
IORT: Intraoperative Radiation Therapy
ISO-TCP: Special low-level network communication protocol based on TCP
IIS: Internet Information Server
Javascript: Scripting language
JQuery: JavaScript library
HIS: Hospital Information System
HYDMEDIA: Heydt Verlag document and image archive
KHP: Vendor of software
LDAP: Lightweight Directory Access Protocol
Linac: Linear accelerator
Linux: Operating system (open source)
MEDATEC: Doctor letters system (IN-TEC)
MiraPlus: Central finding server (HIS)
MOSAIQ: Departmental system (Elekta) R&V-System
MRT: Magnetic Resonance Tomography
MS SQL: Data-management System (Microsoft)
MU: Monitor Units
MySQL: Data-management System (open source)
ODBC: Database API (application interface)
OPS: Operation and procedure codes
PHP: Scripting language
PDV-FR: HIS of hospital centre
PostgreSQL: Data-management System (open source)
Python: Scripting language
RTT: Radiation Therapy Technologist (linac radiographers)
R&V: Record and Verify System
Ruby: Scripting language
TCP: Transmission Control Protocol
Theranostic: Now Elekta
Varian: Vendor of accelerators radiotherapy software, R&V-System
VISIR: R&V-System (THERANOSTIC)
VMAT: Volumetric Modulated Arc Therapy
Windows: Operationg system (Microsoft)
WFM: Workflow Manager (built in tool in MOSAIQ)
